# Supplementary material for: A rapid rise in hormone receptor-positive and HER2-positive breast cancer subtypes in Southern Thai women: A population-based study in Songkhla
Source: PLoS One. 2022 Mar 28;17(3):e0265417. doi: 10.1371/journal.pone.0265417 (PMC8959182; doi:10.1371/journal.pone.0265417)
Supplement: S1 Table — (DOCX) [file pone.0265417.s001.docx]

**S1 Table: Demographics and tumor characteristics of observed data stratified by receptor status**

| **Characteristics** | **Estrogen receptor status** | | | **Progesterone receptor status** | | | **HER2 status** | | |
| --- | --- | --- | --- | --- | --- | --- | --- | --- | --- |
|  | **negative**  **n= 470**  **cases (%)**  **a** | **positive**  **n = 1098**  **cases (%)**  **b** | **unknown**  **n=1315**  **cases (%)**  **c** | **negative**  **n=684**  **cases (%)**  **d** | **positive**  **n = 883**  **cases (%)**  **e** | **unknown**  **n = 1316**  **cases (%)**  **f** | **negative/ equivocal**  **n = 1127**  **cases (%)**  **g** | **positive**  **n = 319**  **cases (%)**  **h** | **unknown**  **n = 1437**  **cases (%)**  **i** |
| **Age** | | | | | | | | | |
| less than 40 | 63 (13.4) | 106 (9.7) | 127 (9.7) | 78 (11.4) | 91 (10.3) | 127 (9.7) | 125 (11.1) | 32 (10.0) | 139 (9.7) |
| 40-49 | 115 (24.5) | 331 (30.1) | 361 (27.5) | 151 (22.1) | 295 (33.4) | 361 (27.4) | 318 (28.2) | 91 (28.5) | 398 (27.7) |
| 50-59 | 155 (33.0) | 341 (31.1) | 378 (28.7) | 233 (34.1) | 263 (29.8) | 378 (28.7) | 354 (31.4) | 111 (34.8) | 409 (28.5) |
| 60-69 | 87 (18.5) | 202 (18.4) | 256 (19.5) | 150 (21.9) | 138 (15.6) | 257 (19.5) | 213 (18.9) | 59 (18.5) | 273 (19.0) |
| 70 and over | 50 (10.6) | 118 (10.7) | 193 (14.7) | 72 (10.5) | 96 (10.9) | 193 (14.7) | 117 (10.4) | 26 (8.2) | 218 (15.2) |
| **Religion** | | | | | | | | | |
| Buddhist | 420 (89.4) | 981 (89.3) | 1107 (84.2) | 608 (88.9) | 792 (89.7) | 1108 (84.2) | 1008 (89.4) | 289 (90.6) | 1211 (84.3) |
| Muslim | 48 (10.2) | 113 (10.3) | 189 (14.4) | 74 (10.8) | 87 (9.9) | 189 (14.4) | 115 (10.2) | 28 (8.8) | 207 (14.4) |
| other | 0 (0.0) | 2 (0.2) | 6 (0.5) | 0 (0.0) | 2 (0.2) | 6 (0.5) | 1 (0.1) | 1 (0.3) | 6 (0.4) |
| unknown | 2 (0.4) | 2 (0.2) | 13 (1.0) | 2 (0.3) | 2 (0.2) | 13 (1.0) | 3 (0.3) | 1 (0.3) | 13 (0.9) |
| **Morphology** | | | | | | | | | |
| ductal | 442 (94.0) | 932 (84.9) | 1002 (76.2) | 625 (91.4) | 748 (84.7) | 1003 (76.2) | 967 (85.8) | 300 (94.0) | 1109 (77.2) |
| lobular | 13 (2.8) | 58 (5.3) | 63 (4.8) | 25 (3.7) | 46 (5.2) | 63 (4.8) | 64 (5.7) | 4 (1.3) | 66 (4.6) |
| mixed | 6 (1.3) | 35 (3.2) | 29 (2.2) | 15 (2.2) | 26 (2.9) | 29 (2.2) | 26 (2.3) | 9 (2.8) | 35 (2.4) |
| others | 5 (1.1) | 56 (5.1) | 32 (2.4) | 13 (1.9) | 48 (5.4) | 32 (2.4) | 58 (5.1) | 3 (0.9) | 32 (2.2) |
| unknown | 4 (0.9) | 17 (1.5) | 189 (14.4) | 6 (0.9) | 15 (1.7) | 189 (14.4) | 12 (1.1) | 3 (0.9) | 195 (13.6) |
| **Grade** | | | | | | | | | |
| well-differentiated | 19 (4.0) | 139 (12.7) | 149 (11.3) | 38 (5.6) | 120 (13.6) | 149 (11.3) | 129 (11.4) | 14 (4.4) | 164 (11.4) |
| moderately- differentiated | 118 (25.1) | 444 (40.4) | 510 (38.8) | 191 (27.9) | 370 (41.9) | 511 (38.8) | 406 (36.0) | 117 (36.7) | 549 (38.2) |
| poorly- differentiated | 260 (55.3) | 302 (27.5) | 299 (22.7) | 341 (49.9) | 221 (25.0) | 299 (22.7) | 376 (33.4) | 144 (45.1) | 341 (23.7) |
| undifferentiated | 3 (0.6) | 0 (0.0) | 5 (0.4) | 3 (0.4) | 0 (0.0) | 5 (0.4) | 0 (0.0) | 3 (0.9) | 5 (0.3) |
| unknown | 70 (14.9) | 213 (19.4) | 352 (26.8) | 111 (16.2) | 172 (19.5) | 352 (26.7) | 216 (19.2) | 41 (12.9) | 378 (26.3) |
| **Stage** | | | | | | | | | |
| local | 63 (13.4) | 256 (23.3) | 130 (9.9) | 105 (15.4) | 214 (24.2) | 130 (9.9) | 260 (23.1) | 36 (11.3) | 153 (10.6) |
| regional | 352 (74.9) | 687 (62.6) | 609 (46.3) | 489 (71.5) | 549 (62.2) | 610 (46.4) | 727 (64.5) | 229 (71.8) | 692 (48.2) |
| distant | 34 (7.2) | 110 (10.0) | 99 (7.5) | 63 (9.2) | 81 (9.2) | 99 (7.5) | 93 (8.3) | 43 (13.5) | 107 (7.4) |
| unknown | 21 (4.5) | 45 (4.1) | 477 (36.3) | 27 (3.9) | 39 (4.4) | 477 (36.2) | 47 (4.2) | 11 (3.4) | 485 (33.8) |
| **Diagnosis year** | | | | | | | | | |
| 2009 | 44 (9.4) | 61 (5.6) | 101 (7.7) | 51 (7.5) | 54 (6.1) | 101 (7.7) | 62 (5.5) | 28 (8.8) | 116 (8.1) |
| 2010 | 34 (7.2) | 69 (6.3) | 124 (9.4) | 45 (6.6) | 58 (6.6) | 124 (9.4) | 84 (7.5) | 8 (2.5) | 135 (9.4) |
| 2011 | 50 (10.6) | 111 (10.1) | 132 (10.0) | 65 (9.5) | 96 (10.9) | 132 (10.0) | 120 (10.6) | 31 (9.7) | 142 (9.9) |
| 2012 | 45 (9.6) | 80 (7.3) | 96 (7.3) | 59 (8.6) | 66 (7.5) | 96 (7.3) | 89 (7.9) | 27 (8.5) | 105 (7.3) |
| 2013 | 42 (8.9) | 99 (9.0) | 128 (9.7) | 61 (8.9) | 79 (8.9) | 129 (9.8) | 107 (9.5) | 21 (6.6) | 141 (9.8) |
| 2014 | 45 (9.6) | 122 (11.1) | 124 (9.4) | 74 (10.8) | 93 (10.5) | 124 (9.4) | 108 (9.6) | 40 (12.5) | 143 (10.0) |
| 2015 | 51 (10.9) | 106 (9.7) | 139 (10.6) | 73 (10.7) | 84 (9.5) | 139 (10.6) | 118 (10.5) | 30 (9.4) | 148 (10.3) |
| 2016 | 51 (10.9) | 149 (13.6) | 154 (11.7) | 78 (11.4) | 122 (13.8) | 154 (11.7) | 147 (13.0) | 36 (11.3) | 171 (11.9) |
| 2017 | 62 (13.2) | 158 (14.4) | 140 (10.6) | 99 (14.5) | 121 (13.7) | 140 (10.6) | 163 (14.5) | 46 (14.4) | 151 (10.5) |
| 2018 | 46 (9.8) | 143 (13.0) | 177 (13.5) | 79 (11.5) | 110 (12.5) | 177 (13.4) | 129 (11.4) | 52 (16.3) | 185 (12.9) |
