# Supplementary material for: A rapid rise in hormone receptor-positive and HER2-positive breast cancer subtypes in Southern Thai women: A population-based study in Songkhla
Source: PLoS One. 2022 Mar 28;17(3):e0265417. doi: 10.1371/journal.pone.0265417 (PMC8959182; doi:10.1371/journal.pone.0265417)
Supplement: S2 Table — (DOCX) [file pone.0265417.s002.docx]

**S2 Table: Demographics and tumor characteristics stratified by receptor status after imputation of unknown receptor status** (columns c, f, i of S1 table)**

| **Characteristics** | **Estrogen receptor status** | | **Progesterone receptor status** | | **HER2 status** | |
| --- | --- | --- | --- | --- | --- | --- |
|  | **negative**  **n = 886***  **median cases (%)** | **positive**  **n = 1997***  **median cases (%)** | **negative**  **n = 1264***  **median cases (%)** | **positive**  **n = 1619***  **median cases (%)** | **negative/equivocal**  **n = 2216***  **median cases (%)** | **positive**  **n = 667***  **median cases (%)** |
| **Age** | | | | | | |
| less than 40 | 108 (12.2) | 188 (9.4) | 132 (10.4) | 164 (10.1) | 235 (10.6) | 61 (9.2) |
| 40-49 | 213 (24.1) | 594 (29.7) | 275 (21.7) | 532 (32.9) | 618 (27.9) | 189 (28.4) |
| 50-59 | 279 (31.6) | 595 (29.8) | 410 (32.4) | 464 (28.7) | 657 (29.6) | 217 (32.6) |
| 60-69 | 165 (18.7) | 380 (19.0) | 283 (22.4) | 262 (16.2) | 419 (18.9) | 126 (18.90 |
| 70 and over | 119 (13.5) | 242 (12.1) | 165 (13.0) | 196 (12.1) | 289 (13.0) | 72 (10.8) |
| **Religion** | | | | | | |
| Buddhist | 765 (86.5) | 1743 (87.2) | 1088 (86.2) | 1420 (87.6) | 1922 (86.7) | 586 (88.1) |
| Muslim | 109 (12.3) | 241 (12.1) | 165 (13.1) | 185 (11.4) | 280 (12.6) | 70 (10.5) |
| other | 0 (0.0) | 8 (0.4) | 0 (0.0) | 8 (0.5) | 3 (0.1) | 5 (0.8) |
| unknown | 10 (1.1) | 7 (0.4) | 9 (0.7) | 8 (0.5) | 13 (0.6) | 4 (0.6) |
| **Morphology** | | | | | | |
| ductal | 780 (88.3) | 1596 (79.8) | 1085 (86.0) | 1291 (79.6) | 1801 (81.2) | 575 (86.6) |
| lobular | 28 (3.2) | 106 (5.3) | 50 (4.0) | 84 (5.2) | 125 (5.6) | 9 (1.4) |
| mixed | 9 (1.0) | 61 (3.1) | 25 (2.0) | 45 (2.8) | 52 (2.3) | 18 (2.7) |
| others | 8 (0.9) | 85 (4.3) | 20 (1.6) | 73 (4.5) | 88 (4.0) | 5 (0.8) |
| unknown | 58 (6.6) | 152 (7.6) | 82 (6.5) | 128 (7.9) | 153 (6.9) | 57 (8.6) |
| **Grade** | | | | | | |
| well-differentiated | 45 (5.1) | 262 (13.1) | 82 (6.5) | 225 (13.9) | 274 (12.4) | 33 (5.0) |
| moderately- differentiated | 253 (28.6) | 819 (41.0) | 392 (31.0) | 680 (42.0) | 815 (36.8) | 257 (38.6) |
| poorly- differentiated | 407 (45.9) | 454 (22.7) | 526 (41.6) | 335 (20.7) | 619 (27.9) | 242 (36.3) |
| undifferentiated | 8 (0.9) | 0 (0.0) | 8 (0.6) | 0 (0.0) | 0 (0.0) | 8 (1.2) |
| unknown | 173 (19.5) | 462 (23.1) | 257 (20.3) | 378 (23.4) | 509 (23.0) | 126 (18.9) |
| **Stage** | | | | | | |
| local | 92 (10.4) | 357 (17.9) | 149 (11.8) | 300 (18.5) | 391 (17.6) | 58 (8.7) |
| regional | 546 (61.8) | 1102 (55.1) | 766 (60.6) | 882 (54.5) | 1254 (56.5) | 394 (59.2) |
| distant | 55 (6.2) | 188 (9.4) | 106 (8.4) | 137 (8.5) | 166 (7.5) | 78 (11.7) |
| unknown | 191 (21.6) | 352 (17.6) | 243 (19.2) | 300 (18.5) | 407 (18.3) | 136 (20.4) |
| **Diagnosis year** | | | | | | |
| 2009 | 84 (9.5) | 122 (6.1) | 100 (7.9) | 106 (6.5) | 152 (6.9) | 54 (8.1) |
| 2010 | 82 (9.3) | 145 (7.3) | 102 (8.1) | 125 (7.7) | 186 (8.4) | 41 (6.1) |
| 2011 | 101 (11.4) | 192 (9.6) | 128 (10.1) | 165 (10.2) | 229 (10.3) | 64 (9.6) |
| 2012 | 79 (8.9) | 142 (7.1) | 102 (8.1) | 119 (7.3) | 170 (7.7) | 51 (7.6) |
| 2013 | 82 (9.30 | 187 (9.4) | 116 (9.2) | 153 (9.4) | 214 (9.7) | 55 (8.2) |
| 2014 | 82 (9.30 | 209 (10.5) | 127 (10.1) | 164 (10.1) | 216 (9.7) | 75 (11.2) |
| 2015 | 91 (10.3) | 205 (10.3) | 132 (10.5) | 164 (10.1) | 229 (10.3) | 67 (10.0) |
| 2016 | 99 (11.2) | 255 (12.8) | 147 (11.6) | 207 (12.8) | 275 (12.4) | 79 (11.8) |
| 2017 | 96 (10.8) | 264 (13.2) | 156 (12.4) | 204 (12.6) | 277 (12.5) | 83 (12.4) |
| 2018 | 89 (10.1) | 277 (13.9) | 152 (12.0) | 214 (13.2) | 268 (12.1) | 98 (14.7) |

* The median of 1,000 imputed datasets

** The cases are the medians of cases from 1,000 imputed datasets with specific demographics and tumor characteristics stratified by receptor status. The Multivariate Imputation by Chained Equations (MICE) package in R-program was used in the imputation. Thus, the unknown receptor statuses in S1B Table were imputed into negative/equivocal or positive status based on the correlation between receptor status and other demographic and histopathologic variables. The percentages are column percent of the characteristics by receptor status.
