# Supplementary material for: A rapid rise in hormone receptor-positive and HER2-positive breast cancer subtypes in Southern Thai women: A population-based study in Songkhla
Source: PLoS One. 2022 Mar 28;17(3):e0265417. doi: 10.1371/journal.pone.0265417 (PMC8959182; doi:10.1371/journal.pone.0265417)
Supplement: S1 Appendix — (DOCX) [file pone.0265417.s003.docx]

**S1 Appendix: The calculated margin of error** **of imputed datasets by three methods**

The mean/median of proportion and margin of error of 1,000 imputed datasets were calculated by three methods, including

- the 95% confidence interval estimated by Rubin’s rule for mean
- the 95% probability interval using the 2SD method for mean
- the 95% probability interval by quantile method for median

Manually calculate the standard error (SE) as the margin of error using Rubin’s rule [1–3] for a single population parameter of interest Q, the multiple imputation (MI) overall point estimate is the average of the m estimates of Q from the imputed datasets. When Q is the proportion (p) of a histologic group,

then $\overline{Q}= \frac{1}{m} \sum_{i=1}^{m} \hat{Q}_{i}$,

the associated total variance for the proportion $T= \overline{U}+\left( 1+ \frac{1}{m} \right)B$,

where $\overline{U}= \frac{1}{m} \sum_{i=1}^{m} U_{i}$ is the estimated within imputation variance, while $U_{i}=\hat{Q}_{i} (1-\hat{Q}_{i})$

and $B= \frac{1}{m-1} \sum_{i=1}^{m} \left( \hat{Q}_{i}- \overline{Q} \right)^{2}$ is the between imputation variance and SE is the square root of the imputation variance = $\sqrt{T}$.

**References**

1. Souverein OW, Zwinderman AH, Tanck MWT. Multiple Imputation of Missing Genotype Data for Unrelated Individuals. Annals of Human Genetics. 2006;70(3):372–81.

2. Yuan Y. Multiple Imputation Using SAS Software. Journal of Statistical Software. 2011 Dec 12;45:1–25. doi: 10.18637/jss.v045.i06.

3. Marshall A, Altman DG, Holder RL, Royston P. Combining estimates of interest in prognostic modelling studies after multiple imputation: current practice and guidelines. BMC Med Res Methodol. 2009 Dec;9(1):57. doi: 10.1186/1471-2288-9-57. PMID: 19638200.

The R script for calculation.

load("imputed datasets.Rdata")

TNBC<-NULL

for (i in 1:1000){

TNBC[i] <-sum(Unk.list[[i]]$ER=="negative" & Unk.list[[i]]$PR=="negative" & Unk.list[[i]]$HER2==0)

}

enrich.HER2<-NULL

for (i in 1:1000){

enrich.HER2[i] <-sum(Unk.list[[i]]$ER=="negative" & Unk.list[[i]]$PR=="negative" & Unk.list[[i]]$HER2==1)

}

lu1<-lu2<-lu3<-lu4<-lu5<-lu6 <-NULL

for (i in 1:1000){

lu1[i] <- sum(Unk.list[[i]]$ER=="negative" & Unk.list[[i]]$PR=="positive" & Unk.list[[i]]$HER2==0)

lu2[i] <- sum(Unk.list[[i]]$ER=="negative" & Unk.list[[i]]$PR=="positive" & Unk.list[[i]]$HER2==1)

lu3[i] <- sum(Unk.list[[i]]$ER=="positive" & Unk.list[[i]]$PR=="negative" & Unk.list[[i]]$HER2==0)

lu4[i] <- sum(Unk.list[[i]]$ER=="positive" & Unk.list[[i]]$PR=="positive" & Unk.list[[i]]$HER2==0)

lu5[i] <- sum(Unk.list[[i]]$ER=="positive" & Unk.list[[i]]$PR=="negative" & Unk.list[[i]]$HER2==1)

lu6[i] <- sum(Unk.list[[i]]$ER=="positive" & Unk.list[[i]]$PR=="positive" & Unk.list[[i]]$HER2==1)

}

lumipp<-NULL

for (i in 1:1000){

lumipp[i] <-sum(lu2[i],lu5[i],lu6[i])

}

lumipn<-NULL

for (i in 1:1000){

lumipn[i] <-sum(lu1[i],lu3[i],lu4[i])

}

### luminal A-like ###

#####################

m <- 1000

All <-2883

##Rubin method

p <- lumipn/All

U <- p*(1-p)/m^2

Ub <- (1/m)*sum(U)

Qb <- 1/m*sum(p)

B <- (1/(m-1)*sum((p-Qb)^2))

T <- Ub + (1+1/m)*B

SE <- sqrt(T)

ave <- sum(p)/m

ave

ave.1 <-median(p)

ave.1

##Rubin

L.Rub <- ave-qnorm(0.975)*SE

U.Rub <- ave+qnorm(0.975)*SE

ave; L.Rub; U.Rub

# quantile method

L.Qua <- quantile(p, 0.025)

U.Qua <- quantile(p, 0.975)

ave.1;L.Qua;U.Qua

# 2SD method

L.2SD <- ave-2*sd(p)

U.2SD <- ave+2*sd(p)

ave;L.2SD;U.2SD

### luminal B-like ###

#####################

m <- 1000

All <-2883

##Rubin method

p <- lumipp/All

U <- p*(1-p)/m^2

Ub <- (1/m)*sum(U)

Qb <- 1/m*sum(p)

B <- (1/(m-1)*sum((p-Qb)^2))

T <- Ub + (1+1/m)*B

SE <- sqrt(T)

ave <- sum(p)/m

ave

ave.1 <-median(p)

ave.1

##Rubin

L.Rub <- ave-qnorm(0.975)*SE

U.Rub <- ave+qnorm(0.975)*SE

ave; L.Rub; U.Rub

# quantile method

L.Qua <- quantile(p, 0.025)

U.Qua <- quantile(p, 0.975)

ave.1;L.Qua;U.Qua

# 2SD method

L.2SD <- ave-2*sd(p)

U.2SD <- ave+2*sd(p)

ave;L.2SD;U.2SD

### Triple-negative ###

#####################

m <- 1000

All <-2883

##Rubin method

p <- TNBC/All

U <- p*(1-p)/m^2

Ub <- (1/m)*sum(U)

Qb <- 1/m*sum(p)

B <- (1/(m-1)*sum((p-Qb)^2))

T <- Ub + (1+1/m)*B

SE <- sqrt(T)

ave <- sum(p)/m

ave

ave.1 <-median(p)

ave.1

##Rubin

L.Rub <- ave-qnorm(0.975)*SE

U.Rub <- ave+qnorm(0.975)*SE

ave; L.Rub; U.Rub

# quantile method

L.Qua <- quantile(p, 0.025)

U.Qua <- quantile(p, 0.975)

ave.1;L.Qua;U.Qua

# 2SD method

L.2SD <- ave-2*sd(p)

U.2SD <- ave+2*sd(p)

ave;L.2SD;U.2SD

### HER2-enriched ###

#####################

m <- 1000

All <-2883

##Rubin method

p <- enrich.HER2/All

U <- p*(1-p)/m^2

Ub <- (1/m)*sum(U)

Qb <- 1/m*sum(p)

B <- (1/(m-1)*sum((p-Qb)^2))

T <- Ub + (1+1/m)*B

SE <- sqrt(T)

ave <- sum(p)/m

ave

ave.1 <-median(p)

ave.1

##Rubin

L.Rub <- ave-qnorm(0.975)*SE

U.Rub <- ave+qnorm(0.975)*SE

ave; L.Rub; U.Rub

# quantile method

L.Qua <- quantile(p, 0.025)

U.Qua <- quantile(p, 0.975)

ave.1;L.Qua;U.Qua

# 2SD method

L.2SD <- ave-2*sd(p)

U.2SD <- ave+2*sd(p)

ave;L.2SD;U.2SD
