# Supplementary material for: A rapid rise in hormone receptor-positive and HER2-positive breast cancer subtypes in Southern Thai women: A population-based study in Songkhla
Source: PLoS One. 2022 Mar 28;17(3):e0265417. doi: 10.1371/journal.pone.0265417 (PMC8959182; doi:10.1371/journal.pone.0265417)
Supplement: Attachment — Submitted filename: Response to reviewers.docx [file pone.0265417.s004.docx]

**RESPONSE TO EDITOR AND REVIEWER**

RESPONSE TO THE EDITOR:

***MAJOR POINTS:***

**COMMENT:** I would suggest authors to add more details about the cancer registry and how a cancer case is defined. Could you tell be more about the population covered by the registry? Does it cover all the province? The authors mentioned that the registry reports « both population-based and hospital-based statistics ». This is unclear. How do a cancer case is defined? What is the date of incidence considered? How did authors consider cancer diagnosis based on death certificate only (DCO)?

RESPONSE: We added more detail about the Songkhla cancer registry and revised the unclear sentences (Study setting and data sources paragraph: 3-4).

“The Songkhla Cancer Registry is a population-based cancer registry that enrolled cancer patients residing in Songkhla Province. Case ascertainment began in 1988, and patients’ data were regularly supplied to the Cancer Incidence in Five Continents (CIV since vol. VIII) [15]. The cases were identified using active and passive case-finding methods and registered by trained staff. The sources of information included medical records and pathological records of all hospitals in the province. The status of the patients in the database is reported when deaths occur in the hospitals and healthcare network and is confirmed by the Bureau of Registration Administration Database, Department of Provincial Administration, Ministry of Interior.

The diagnosis date of cancer patients was obtained from the most reliable source, if possible. The diagnosis date was indicated as the date of biopsy, the date when the specimen was received at the pathological laboratory, or the report date. When the date of definitive diagnosis provided by the pathological laboratory was not indicated, the date of hospital admission due to this malignancy and other dates related to the occurrence of malignancy or death were considered [16].”

**COMMENT:** In this analysis, did authors apply an age criteria for inclusion?

RESPONSE: We included all breast cancer cases who were diagnosed in the study periods. However, from our data, the youngest patient who met our criteria was 24 years old. That is why we set the age group for calculating the age-specific incidence from age 20 to ≥85 years.

**COMMENT:** Also, I really like the discussion part about number of new cancer cases and estimated costs (lines 335-344). This would deserve to be part of the results part and to be presented as a secondary objective. This is an interesting point that may be of interest for policy makers. I would highlight that point. If authors decide to do so, they need to explain about they estimate the nb of new cancer cases in 2030 and how they estimate the excess costs in the method section.

RESPONSE: We decided to set the projection of additional cost due to the trastuzumab treatment in 2030 as the secondary objective of our study. We explained how to estimate the excess cost in the methodology part as subtopic “Trastuzumab cost projection” and added the outcome in the result part under the subtopic “Additional cost projection of trastuzumab treatment.”

**COMMENT:** Why did not the authors use the Rubin's rules to combine their estimates?

RESPONSE: In our analysis, we calculated the estimates and margin of error of the imputed data by three methods, median and its 95% probability interval, mean, and Rubin’s estimate of the 95% confidence interval and mean ±2 SD margins. All three methods gave very close values of the estimates and the margins of error. Thus, in the previous draft, we decided to show only the median proportion and 95%PI, but we forgot to explain the similarity of the results. So, in this revision, we showed the results from all methods in Table 2. (Table 2 and Results part in subtopic: Proportions of breast cancer M-IHC subtypes)

**COMMENT:** Because authors studied trends in cancer incidence, I would like to see a comment in the discussion on the possible change (or not) of recording system of new cancer cases. Also, authors showed that there is a decrease in incidence from the age of 70. Would not it be explained by a lower ascertainment of cancer cases at older ages? Older adults may be less likely to go through cancer diagnosis workups because of their age, the presence of other conditions, or frailty. This should be mentioned as possible reason.

RESPONSE: One study from six provincial cancer registries in Thailand found an effect of introducing a country-wide self-breast examination screening program under the universal health insurance, which covered over 90% of the Thai population increase capturing of the hidden breast cancer cases aged over 80. The capture rate declined after some years of the program's start even it has continued as many cases have been captured. So, we think that we captured the decline in the ascertainment rate during our study period and is becoming stabilized after the end of our study. In the paragraph, we discussed that “Thailand has promoted breast cancer screening programs since 2002, covering over 90% of the Thai population [3]. An analysis of six provincial cancer registries in Thailand reported the effect of introducing a country-wide self-breast examination screening program [3]. It increased the detection of breast cancer among individual aged >80 years. However, the detectable rate declined several years after that because few prevalent cases were reported. Our study also found a decline in the age-specific incidence rates of all breast cancer subtypes after the age of 70 years, which is consistent with the report of a previous study.” (Discussion: paragraph 3).

**COMMENT:** Another possible reason for changes in incidence is breastfeeding practices? Have they changed over the period covered by the study?

RESPONSE: We added the detail about the change in the breastfeeding rate in Thailand over time in the revised version (Discussion: paragraph 4).

“However, the 6-month exclusive breastfeeding rate in Thailand has changed over time. A series of national surveys reported that the rate was 14.5% in 2005 and increased to 23.1% in 2016, which was the highest; however, it sharply decreased to 14.0% in 2019 [27].”

27.	Topothai C, Tangcharoensathien V. Achieving global targets on breastfeeding in Thailand: gap analysis and solutions. Int Breastfeed J. 2021 Dec;16(1):38. doi: 10.1186/s13006-021-00386-0. PMID: 33962645.

***MINOR POINTS:***

**COMMENT:** I would suggest to replace 10e5 by 100,000 as it is easier to read.

RESPONSE: We agree with the editor's comment and change text "10^5^" to "100,000" in our revised version.

**COMMENT:** I strongly encourage authors to have an English-native speaker to check the grammar and sentence structure as there are several parts of text that are unclear. For instance, lines 223-231, 243, 259, 264-266, 196-299, 331-333, 355-359, 363. I encourage authors to reformulate.

RESPONSE: Before resubmission, we sent the revised manuscript to the Editage for checking the grammar and sentence structure.

**COMMENT:** The introduction may be shortened, in particular, the paragraph on racial and cultural disparities. Because the study is about incidence, the paragraph on treatment does not seem relevant in the introduction.

RESPONSE: We decided to delete the paragraph that describes the racial variations in Thailand. However, we remained the paragraph that described the treatment option in breast cancer patients. It shows why the knowledge of breast cancer incidence in terms of subtype is essential. In addition, it relates to our secondary objective that we added in the revised version.

**COMMENT:** Authors mentioned cancer stage several times but they did not define which staging system they used. I would then suggest to add the definition in the method section.

RESPONSE: Songkhla cancer registry records the breast cancer stage according to the AJCC-TNM classification. In a long-running cancer registry, cancer staging may change over time. The registries usually reclassify the extent of disease to the summary stage according to the SEER staging system. It categorized tumor stage to in situ, localized, regionalized, distant, and unknown stage.

**COMMENT:** Lines 304-305: is not tautological?

RESPONSE: We revised the sentences in Line 304-305 and described this issue in more detail as “However, the 6-month exclusive breastfeeding rate in Thailand has changed over time. A series of national surveys reported that the rate was 14.5% in 2005 and increased to 23.1% in 2016, which was the highest; however, it sharply decreased to 14.0% in 2019 [27].” (Discussion: paragraph 4).

**COMMENT:** Lines 311-312: authors mentioned an increase in teenage pregnancy. Would not it explain by a better reporting?

RESPONSE: We changed the last part of the discussion on reproductive behaviors in the Thai population to lessen the stress on teenage pregnancy and HR-positive breast cancer. The detail is, “Thai adolescents experienced a social dynamism in reproductive behavior, which possibly interfered with the overall change in internal estrogen exposure in this age group. From 2000 to 2012, the rate of teenage pregnancy has been increasing in Thai society [31], while the abortion rate due to unwanted pregnancies is high [32]. Such social effects on the reproductive age might have contributed to the incidence of HR-positive breast cancer.”

In addition, we also changed reference no. 31 (previous version no.29) to “Sukrat B. Thailand Adolescent Birth Rate: Trend and Related Indicators. Thai J Obstet Gynaecol. 2014 Jan 20;15–21”. (Discussion: paragraph 5)

**COMMENT:** Lines 315-317: I would invite caution with this interpretation.

RESPONSE: We revised the sentences, described them in more detail. The new text is “Exogenous hormone exposure, particularly combined estrogen/progesterone hormone replacement therapy, may have also positively increased the risk of HR-positive subtypes [33,34]. Two studies on female hormone use in Thailand demonstrated no association between exogenous hormonal use and breast cancer risk. However, these two studies did not specify the HR subtype of breast cancer [35-36].”

In addition, we changed the reference to “Type and timing of menopausal hormone therapy and breast cancer risk: individual participant meta-analysis of the worldwide epidemiological evidence. The Lancet. 2019 Sep;394(10204):1159–68”. (Discussion: paragraph 6)

**COMMENT:** Lines 317: Are not there any data at all?

RESPONSE: Of course. We found only a few studies [35,36] on female hormone use and breast cancer risk. However, they are not specific on breast cancer subtypes or receptor status. They did not find the association between hormone use and breast cancer risk. We described more in the revised version. (Discussion: paragraph 6)

35. Poosari A, Promthet S, Kamsa-ard S, Suwanrungruang K, Longkul J, Wiangnon S.	Hormonal Contraceptive Use and Breast Cancer in Thai Women. J Epidemiol. 2014	May 5;24(3):216–20.

36.	Ratanawichitrasin A, Bhodhisuwan K, Reansuwan W, Kongpatanakul S, Ratanawichitrasin S. Risk of Breast Cancer in Post-Menopausal Women Using Hormone Replacement Therapy. J Med Assoc Thai. 2002 May;85(5):583-9.

**COMMENT:** Lines 320-323: Could authors include some figures in the text? What are the prevalences?

RESPONSE: We added more detail to the paragraph that described the BMI and positive-breast cancer risk and prevalence of obesity in Thai women as “The national surveys reported that the prevalence of overweight and obesity (BMI ≥ 25 mg/m^2^) among Thai women has increased [39-41]. The prevalence increased from 25.1 in 1994 to 34.4 and 41.8 kg/m2 in 2004 and 2014, respectively. In comparison, the prevalence trends of overweight and obesity in the Southern Thai women were 25.4, 36.3, and 43.7 kg/m^2^ in the same three years, respectively. Thus, the increasing trends of BMI in southern Thai women seemed to be higher than the average BMI of Thai women. This may partly explain the rising trends in HR-positive breast cancer in Thailand.” (Discussion: paragraph 7).

**COMMENT:** Figures: I would recommend to add confidence intervals around curves.

RESPONSE: We added the 95% confidence interval in Fig 1 following the editor’s suggestion and change y-scale from log- to normal scale.

**COMMENT:** Authors used both relative risk and rate ratios. I advise authors to pick one and stick with it throughout the manuscript. However, relative risk and rate ratios are not similar. Authors should probably clarify which one is estimated by APC models.

RESPONSE: We already clarified that the RR is the rate ratio like we notice in figure 2. We already revised the error text in our manuscript.

**COMMENT:** Line 109: what means stage 0 or extent 1?

Line 118: authors wrote « [the registry] collects cancer patients diagnosed or treated ». This is unclear. How does the cancer registry collect data on new cancer cases?

Line 161: Which period did authors consider as « recent »?

RESPONSE: Line 109: We included only invasive tumors, added “invasive” (subtopic: Study design and participants, paragraph 2) So, we can delete “in situ tumor (code as stage 0 or extent 1)”.

Line 118: We already added the detail about the cancer registry. (Setting and data sources: paragraph 3-4).

Line 161: we specified the period when we used the coefficients to project the future trend “coefficients from 2009-2018”. (Subtopic: Incidence rates, trends, and projection, paragraph 4)

**RESPONSE TO REVIEWER 1:**

**COMMENT:** The authors present the first population-based cancer registry data to summarize molecular-immune-histochemical subtypes. Overall, the authors provide a very rigorous analysis to interpret trends in hormone receptor status of primary breast cancer cases from 2009 to 2018. This is an important research topic, but I have some concerns, mainly related to the level of missing in the data.

RESPONSE: Our analysis found high percentages of missing data on ER, PR, and HER2 status, 45.6, 45.6, and 49.8%, respectively. Literature reviews found that multiple imputation could reduce bias and improved effect estimation at the high proportion of missing data. It is especially when imputation was conducted based on the auxiliary information, including variables predicting the complete set of the hormone and HER2 receptor statuses in the model. Thus, the replacement of the cases with missing values of the ER, PR, and HER2 statuses was random and conformed to the original construct of the association of the non-missing cases. The maximum of missing proportions that can hold by the multiple imputation method is different. However, the evidence shows that the multiple imputation by chained equation (MICE) method can handle up to 80% of missingness [3].

References.

1.	Eisemann N, Waldmann A, Katalinic A. Imputation of missing values of tumour stage in population-based cancer registration. BMC Med Res Methodol. 2011 Dec;11(1):129.

2.	Madley-Dowd P, Hughes R, Tilling K, Heron J. The proportion of missing data should not be used to guide decisions on multiple imputation. J Clin Epidemiol. 2019 Jun;110:63–73.

3.	Souverein OW, Zwinderman AH, Tanck MWT. Multiple Imputation of Missing Genotype Data for Unrelated Individuals. Annals of Human Genetics. 2006;70(3):372–81.

**COMMENT:**

Introduction

I would suggest a little reorganizing of this paragraph "Since 2002, Thailand has implemented the universal health coverage (UHC) scheme [10]. It is the basic scheme for Thai citizens, covered health promotion, disease screening, treatment of basic and high-cost diseases under the Thai national list of essential medicines 5 (NLEM), and palliative/supportive care. The UHC scheme contributed approximately 79% of the Thai population's health insurance [11].".

Mainly, I would recommend mentioning that 79% of the population's health insurance is the basic covered should be mentioned after the fact it is the basic scheme for Thai citizens. I would also recommend switching covered to covers as the UHC appears to still be in place.

RESPONSE: We revised the sentence sequencing in the paragraph, “Since 2002, Thailand has implemented a universal health coverage (UHC) scheme [7]. This is the primary insurance scheme for Thai citizens. As of 2020, the UHC scheme has already provided health services to approximately 79% of the Thai population [8]. It covers health promotion, disease screening, treatment of basic and high-cost diseases under the Thai National List of Essential Medicines (NLEM), and palliative/supportive care.” following the suggestion from the reviewer. (Introduction: paragraph 3)

**COMMENT:**

The authors stated "Though a targeted therapy is cost-effective among early breast cancer cases, it increases the burden of cancer care costs as a whole and significantly contributes to the national health budget, especially when early patients are more detected ".

I am not exactly following your logic on how it would significantly increase the burden of cancer care costs as a whole if it is a cost-effective treatment. Can you please elaborate?

The authors state "Other targeted therapeutic drugs for breast cancer have been or are in Thai FDA". Can you please provide a few examples?

RESPONSE:

Though a targeted therapy is cost-effective among early breast cancer cases, it increases the sum of cancer care budget by 15560 USD per one patient to add a targeted therapy to the to the baseline treatment while the QALYs were expected to increase by 4.59 years. While an increased burden to the national health budget especially when early patients are more detected, in turn, it implies that the longer life gain to women benefited from a targeted therapy would make a return in terms of the GDP per capita greater than the budget spent by the UC scheme for the treatment cost per patient.

In addition, we added information about the other targeted drug that may include in the Thai national list of essential medicines (NLEM) in the future (Introduction: paragraph 6). It is the reason to show that why the estimated incidence in each breast cancer subtype is essential. The additional sentences are “Other targeted drugs approved by the Thai Food and Drug Administration for breast cancer treatment include pertuzumab and ribociclib. Currently, these are the only two drugs available to patients who are covered by the civil servant medical benefit scheme [12,13].”

**COMMENT:**

Methods

The authors mention that they excluded DCIS cases. Yet, DCIS can also be receptor-positive. Can the authors explain why this did not include this group?

RESPONSE: In our population-based cancer registry of Songkhla, it was not designed to collect in situ cases of breast cancer from the start of the registration since the standard of data collection for DCIS tumors is nonhomogeneous in hospitals in the province. It is possible in the future to collect this cancer at the best completeness after the province-wide data collection procedures are standardized.

**COMMENT:**

Management of missing data

The authors report using multiple imputation to account for missing outcome data, but more information would be valuable. Specifically, you mention that the missing in other variables is 22%, but what is the missing for your receptor statuses? This seems like a much bigger concern. It is also important to know how much change this imputation process had on your findings. Mainly, what does the results look like in the non-imputed dataset? How different is the population? I am also a little confused by the process. Did you enter the other missing variables as missing or did you use the imputed predictor variables to determine?

RESPONSE: In our study, we imputed only the missing values in outcome variables, which are ER, PR, and HER2 status. The proportion of missing values in each variable were 45.6, 45.6, and 49.8%, respectively. For the question that which of the missing values were addressed in the imputation, we transformed variables other than the three receptor statuses to factors and set the missing values to an ‘unknown’ class of that variable, so that the ‘unknown’ values have a predictive ability on the receptor statuses in the imputation. We also used the values of one of the three receptor statuses to predict the missing values of the other missing receptor statuses. The final set of the dataset consisted of 1000 imputed data set, a part of them were the known values from the original data, another part of them are imputed data filled into the missing (blank) values.

In Table 1, we added the characteristics of ER, PR, and HER2 status. For example, the numbers of HER2 negative/equivocal, positive, unknown were 1127, 319, 1437, after imputation, the mean of 1000 imputed data set were 2214, 669, and 0, while the SD of the imputed dataset were 38, 38 and 0. After we got the imputed values of the three receptor statuses, the Table 2 described the mean/median of the observed and imputed data with the range of prediction by the three methods. The difference in the observed and imputed proportion is on the third or second decimal places.

In addition, in our revised manuscript, we added the comparison baseline characteristics between observed and imputed data, as the Table 1 in the result part, and added a new table in the supplement (S1A and S1B Table).

**COMMENT:**

Results

Can you please include the range of age at breast cancer diagnosis in addition to the IQR?

RESPONSE: The range of age was 24-95 years. We added the range of age in table1.

**COMMENT:**

Results

It would also be good to see some comparison of demographics by HR status.

From reading the results section, it appears that almost 50% of the receptor status was missing. This is of great concern, especially given that there was an additional 22% of other missing variables. There really needs to be comparisons in the outcome and characteristics of those with and without receptor status, and the new sample with the imputed data to understand the impact.

It is great to see that you included observed data versus imputed data in the results section. I would ask that you include percent in the column "Number of cases". I still think there needs to be a comparison of how the demographics of the women looked before and after imputation.

RESPONSE: We added the percentage in column "Number of cases" of Table 2. The detail about the missing proportion and how we imputed the missingness is described in the previous comment. In addition, we added the baseline characteristic comparison between observed and imputed data according to the comment in table 1 and additional 2 tables (S1A S1B Table) in the supplement.

**COMMENT:**

Discussion

The sentence "In contrast, reproductive factors have an inconclusive influence on HR-negative cancers." Requires a reference

RESPONSE: We decided to revise this paragraph and delete the sentence according to the editor’s comment, making it a clearer comparison. From “In contrast, reproductive factors have an inconclusive influence on HR-negative cancers.” to “The reproductive characteristics of Thai women have been changing due to exposure to higher endogenous estrogen, such as younger age at menarche [28], later age at the first marriage [29], and a fall in the total fertility rate [30] from 4.9 in 1974–1976 to a fertility replacement rate of 2.1 in 1990 and down to 1.6 in 2011. These phenomena may explain the rise in the incidence of HR-positive breast cancer. Thai adolescents experienced a social dynamism in reproductive behavior, which possibly interfered with the overall change in internal estrogen exposure in this age group. From 2000 to 2012, the rate of teenage pregnancy has been increasing in Thai society [31], while the abortion rate due to unwanted pregnancies is high [32]. Such social effects on the reproductive age might have contributed to the incidence of HR-positive breast cancer.” (Discussion: paragraph 5)
