# Supplementary material for: A rapid rise in hormone receptor-positive and HER2-positive breast cancer subtypes in Southern Thai women: A population-based study in Songkhla
Source: PLoS One. 2022 Mar 28;17(3):e0265417. doi: 10.1371/journal.pone.0265417 (PMC8959182; doi:10.1371/journal.pone.0265417)
Supplement: Attachment — Submitted filename: Response to Reviewers .docx [file pone.0265417.s005.docx]

**Response to Reviewers**

Comment for PONE-D-21-21989R1

**Journal Requirements:**

**Suggestion:** Please review your reference list to ensure that it is complete and correct. If you have cited papers that have been retracted, please include the rationale for doing so in the manuscript text, or remove these references and replace them with relevant current references. Any changes to the reference list should be mentioned in the rebuttal letter that accompanies your revised manuscript. If you need to cite a retracted article, indicate the article’s retracted status in the References list and also include a citation and full reference for the retraction notice.

**Response:**

*In manuscript version 2 (PONE-D-21-21989R1), revised from manuscript version 1(PONE-D-21-21989):*

1. We removed the references from manuscript version 1 because the previous references were not relevant after we revised the text in the manuscript. The lists of removed references are as:

- Hays J. People of Thailand: origin, different Thai groups and Siamese twins | facts and details. [cited 15 Jun 2021]. Available from: http://factsanddetails.com/southeast-asia/Thailand/sub5_8c/entry-3209.html
- Srisontisuk DS, Katchamat P, Pakdee P. Poverty and the ethnic minority groups in Thailand. J Mekong Society. 2005;1: 151–189.
- Reid LA. Benedict’s Austro-Tai hypothesis—an evaluation. Asian Perspect. 1984;26: 19–34.
- Aekplakorn W, Inthawong R, Kessomboon P, Sangthong R, Chariyalertsak S, Putwatana P, et al. Prevalence and trends of obesity and association with socioeconomic status in Thai adults: national health examination surveys, 1991–2009. J Obes. 2014;2014: 410259. doi:10.1155/2014/410259. PMID: 24757561.
- Sakboonyarat B, Pornpongsawad C, Sangkool T, Phanmanas C, Kesonphaet N, Tangthongtawi N, et al. Trends, prevalence and associated factors of obesity among adults in a rural community in Thailand: serial cross-sectional surveys, 2012 and 2018. BMC Public Health. 2020;20. doi:10.1186/s12889-020-09004-w. PMID: 32493314.

2. We added some references in manuscript version 2 because we added the new text in manuscript 2. The lists of added references are as:

- Drug and Medical Supply Information Center, Ministry of Public Health. Medical Reimbursement Criteria for Cancer and Hematology Patients Who Need Expensive Drugs (Additional) (W 339, W 340) [Internet]. [cited 2021 Oct 29]. Available from: http://dmsic.moph.go.th/index/detail/7823
- Drug and Medical Supply Information Center, Ministry of Public Health. Medical Reimbursement Criteria for Cancer and Hematology Patients Who Need Expensive Drugs (Additional) (W 278, W 279) [Internet]. [cited 2021 Oct 29]. Available from: http://dmsic.moph.go.th/index/detail/8221
- Bray F, Colombet M, Ferlay J, Mery L, Piñeros M, Znaor A, et al. Cancer Incidence in Five Continents Volume XI [Internet]. [cited 2021 Oct 31]. Available from: https://publications.iarc.fr/Book-And-Report-Series/Iarc-Scientific-Publications
- European Network of Cancer Registries (ENCR).pdf [Internet]. [cited 2021 Oct 31]. Available from: https://www.encr.eu/sites/default/files/pdf/incideng.pdf
- Topothai C, Tangcharoensathien V. Achieving global targets on breastfeeding in Thailand: gap analysis and solutions. Int Breastfeed J. 2021 Dec;16(1):38. doi: 10.1186/s13006-021-00386-0. PMID: 33962645.
- Poosari A, Promthet S, Kamsa-ard S, Suwanrungruang K, Longkul J, Wiangnon S. Hormonal Contraceptive Use and Breast Cancer in Thai Women. J Epidemiol. 2014 May 5;24(3):216–20.
- Ratanawichitrasin A, Bhodhisuwan K, Reansuwan W, Kongpatanakul S, Ratanawichitrasin S. Risk of Breast Cancer in Post-Menopausal Women Using Hormone Replacement Therapy. J Med Assoc Thai. 2002 May;85(5):583-9.
- Thai National Health Examination Survey 1994-1995, NHES I [Internet]. 1st ed. Nonthaburi: Health Systems Research Institute (HSRI); 1996 [cited 2021 Oct 31]. 271 p. Available from: https://www.hiso.or.th/hiso/picture/reportHealth/report/report5.pdf
- Porapakkham Y, Boonyaratapan P, editors. Thai National Health Examination Survey 2003-2004, NHES III [Internet]. 1st ed. Nonthaburi: Health Systems Research Institute (HSRI); 2006 [cited 2021 Oct 31]. 267 p. Available from: https://www.hiso.or.th/hiso/picture/reportHealth/report/report2.pdf
- Aekphakorn W, Pakjaroen H, Thaikla K, Satheannoppakao W. Thai National Health Examination Survey 2013-2014, NHES V [Internet]. 1st ed. Nonthaburi: Health Systems Research Institute (HSRI); 2016 [cited 2021 Oct 31]. 283 p. Available from: https://www.hiso.or.th/hiso/picture/reportHealth/report/report9.pdf

3. We change some of the references from manuscript version 1 as following lists:

- Ref no 31. was changed from *“UNFPA Thailand. [Where do teen mothers live in Thailand?]. [cited 22 May 2021]. Available from: https://thailand.unfpa.org/en/publications”* to *“Sukrat B. Thailand Adolescent Birth Rate: Trend and Related Indicators. Thai Journal of Obstetrics and Gynaecology. 2014 Jan 20;15–21.”*.

The new reference was written in English, while the previous one was written in Thai. However, the information was similar.

- Ref no 34. changed from *“Kim S, Ko Y, Lee HJ, Lim J. Menopausal hormone therapy and the risk of breast cancer by histological type and race: a meta-analysis of randomized controlled trials and cohort studies. Breast Cancer Res Treat. 2018;170: 667–675. doi:10.1007/s10549-018-4782-2. PMID: 29713854.”* to *“Collaborative Group on Hormonal Factors in Breast Cancer. Type and timing of menopausal hormone therapy and breast cancer risk: individual participant meta-analysis of the worldwide epidemiological evidence. The Lancet. 2019 Sep;394(10204):1159–68. doi: 10.1016/S0140-6736(19)31709-X. PMID: 31474332.”.*

The new reference is more relevant and more recent than the previous reference.

*In manuscript version 3, (revised from manuscript version 2; PONE-D-21-21989R1):*

We checked our references (from manuscript version 2). We revised the URL of reference no.18 from *“http://web.nso.go.th/en/census/poph/cen_poph.htm”* to *“http://songkhla.old.nso.go.th/nso/project/search/index.jsp?province”* because the previous URL cannot access presently. However, the information is the same as with the previous reference.

We revised the format of Ref no. 12, 13, 14, 40, 41, and 49 as the mark in “Revised Manuscript with Track Changes” file.

**Additional Editor Comments:**

**Suggestion:** I would advise deleting "and encourage all oncologists and health policymakers to manage breast cancer cases in Thailand." in line 20 as it seems not the right place to make this call.

Line 119: April 20, 2020, October 18, 2020. —> April 20, 2020,  **to** October 18, 2020.

Line 125: The status of the patients —> the **vital** status of the patients

Line 128: The diagnosis date of cancer patients —> **The date of incidence**.

**Response:** We already revised the text in line No. 20, 119, 125 and 128 following the editor suggestion. *(Revised version: Line 20, 119, 125, 129, and 130)*

**Suggestion:** Lines 129-130: Because all these dates may not be the same, please, could you be more specific on how you choose the date? The earlier available?

**Response:** The incidence date was specified following the diagnosis date of cancer patients in the cancer registry database. The date of incidence was obtained from the most reliable source. The incidence date was firstly indicated as the date of biopsy, if not available, the date when the specimen was received at the pathological laboratory, or the report date. When the date of definitive pathological diagnosis was not indicated, the date of hospital admission due to this malignancy, radiological diagnosis, and other clinical diagnosis dates related to the occurrence of malignancy or death were considered. We already revised the paragraph. *(Materials and Methods: Study setting and data sources, paragraph: 4)*

**Suggestion:** Line 137: Does it means that the cancer registry can include patients from other province too? If so, the population covered is not only that of the Songkhla Province. Am I right?

**Response:** The Songkhla cancer registry is a population-based cancer registry. It includes only the cancer patients who are citizens of Songkhla province. The patients are identified from all hospitals in Songkhla province. Songklanagarind hospital is a hospital in Songkla province. The cancer patients of Songklanarind hospital who are the Songkhla citizen are registered to the Songkhla cancer registry and the hospital-based cancer registry of Songklanagarind hospital. While cancer cases, who are not Songkhla citizens, are not registered in the Songkla cancer registry. They are registered only in the hospital-based cancer registry of Songklanagarind hospital. We added more detail as “The hospital has been running both hospital-based CR the Songklanagarind hospital and population-based CR of Songkhla province. Subjects in this study were extracted from the population-based cancer registry.” *(Materials and Methods: Study setting and data sources, paragraph: 5)*

**Suggestion:** Line 431: what do you mean? Missing at random?

**Response:** Yes, we mean the missing at random. We already revised the sentence to more clear as “…In the MICE process, the missing process does not violate the missing at random assumption…”. *(Discussion, paragraph 10)*

**Suggestion:** Line 435: I am not sure to understand the link between this sentence and the following one.

**Response:** Southern Thai women slightly differ from women in other regions regarding genetic and cultural backgrounds. Though, we thought that the inference of this study to all Thai women was still relevant with no solid evidence to validate it.

**Reviewers' comments:**

**Suggestion:**

Introduction:
Please add a reference to the first line “Breast cancer is the most common cancer among women worldwide”.

**Response:** We already added the reference to that sentence. We wrote the sentence based on reference number 1. *(Introduction, paragraph: 1)*

**Suggestion:**
Methods:
If you one method to determine malignancy is based on death, wouldn’t this mean there may be an underestimation of the true prevalence of the disorder? I assume not everyone who dies undergoes an autopsy. This should be addressed in the limitations section.

**Response:** We would like to apologize not to address the diagnosis based on death certificate in the limitation section.

The percentage of the death certificate only (DCO) cases of Songkhla cancer registry was reported around 0.5% in the Cancer Incidence in Five Continents (CI5). Usually, the International Agency for Research on Cancer (IARC) doubts the quality of data when the DCO is zero as the registry might collect cases from pathology sources only. And none of the diagnosis was from autopsy. We are not sure how much pathology sources only bias occurred in diagnosis of breast cancer in the registry. However, the inclusion of cases diagnosed from death certificates ensures a better estimate of the incidence. The cancer registries model the prevalence of cancer based on the incidence and survival, not the count of existing cases in the registry. Since we were estimating the incidence of breast cancer by receptor status, the presence of DCO in cancer diagnosis would not affect the imputation process.

Reference: the International Agency for Research on Cancer (IARC). Indices of data quality of the Cancer Incidence in Five Continents (CI5) volume X. [cited 4 Feb 2022]. Available from: https://ci5.iarc.fr/CI5I-X/old/vol10/I_09.pdf

**Suggestion:**
You mention that your youngest case of breast cancer is in a women who is 24 years of age, yet use 5 year age groups to calculate the age-specific rates. What is the number of individuals who are 24 years of age? This group is likely very small, and unstable. How does the estimate change if you collapse the 24 year old into the upper age group?

**Response:** The number of individuals who are 24 years of age, were 2 cases from 2,883 cases. It estimated 0.1% of total cases.

The age-specific rates were calculated to the unit of cases per 100,000 population in a particular 5-year age group according to Boyle P. & Parkin D.M. in which age-specific rates were calculated from age group of 0–4 to 85+, which the formula as following.

$a_{i}= \left( {r_{i}}/{n_{i}} \right)\times100 000$

Where a_i_ is an age-specific rate per 100,000 population in each five-year age group, r_i_ is the number of cases in the same five-year age group, and n_i_ is the corresponding person-years of the observation.

The ultimate aim of stratifying into five-year strata is to calculate the age-standardized incidence rates (ASR) from the formula as follows:

$ASR= {\sum_{i=1}^{A} a_{i}w_{i}}/{\sum_{i=1}^{A} w_{i}}$

$Var \left( ASR \right) = {\sum_{i=1}^{A} [a_{i}w_{i}^{2}(100 000-a_{i})/n_{i}]}/{{(\sum_{i=1}^{A} w_{i})}^{2}}$

Where a_i_ is an age-specific rate per 100,000 population in each five-year age group, w_i_ is world standard population in the corresponding five-year age group, and n_i_ is the person-years of sample population in the same five-year age group.

The variance of age-specific rate was still low in very young age groups in which the number of patients were small, while the person-years in the sample population were large, see Figure 1.

Reference: P. Boyle and D. M. Parkin, “Statistical Methods for Registries,” In: O. M. Jensen, D. M. Parkin, R. MacLennan, C. S. Muir and R. G. Skeet, Eds., Cancer Registration: Principles and Methods, IARC Scientific Publication No. 95, International Agency for Research on Cancer, Lyon, 1991, pp. 126-158.

**Suggestion:**
I was a little confused by this statement “including our projection of age-specific incidence rates of the HER2- 194 positive subtype in 18 age groups (0–4, 5–9, 10–14, ..., 80–84, and ≥85 years)”. How is this possible if you have the first case of cancer starting at 24 years? Wouldn’t the relative cost for each of these other groups be zero?

**Response:** The age-specific rates were calculated as described above. Even the age-groups with no case have their associated person-years (n_i_) and the 95%CI could be calculated by the following formula

$ASR \pm Z_{\propto/2}\times$ (s.e.(ASR))

$$s.e.\left( ASR \right)= \sqrt{Var (ASR)}$$

The 95%CIs of age-specific rate in the very young age groups were narrow, since the number of patients were small, while the person-years in the sample population were large.

**Suggestion:**
Results:
Please include range of age in this sentence “The median age of the patients at diagnosis of breast cancer was 53.0 years 237 (interquartile range, IQR: 46.0–62.0)”.
**Response:** We already included the age range in this sentence. The new sentence is “The median age of the patients at diagnosis of breast cancer was 53.0 years (interquartile range, IQR: 46.0–62.0; range: 24–95),…” *(Result: Participants and characteristics, paragraph: 2).*

**Suggestion:**
Please also extend the table for age to show each 10 year age group from 20 to give an idea of the instability in estimates for the younger age groups.

**Response:** We already revised table1 to show each 10 ages group, starting from 20-29, 30-39, until 70 and over. *(Table 1)*

**Suggestion:**
Figure 3 title says 105, please change to 100,000. Same in the sentence “Over the study period, the ASRs of other subtypes were not greater than 5.5 cases per 105 women” And “. The ASR of this subtype in 2009 was 2.6 per 105 women.” And here “The overall ASR of HR-positive and HER2-positive (HR+/HER2+ and HR-/HER2+) breast cancer increase from 30-32 and 10-11 to 39 and 13-14 per 105 women in 2020 to 2030”

**Response:** We did not find the sentence that that reviewer suggested to revise in the second version (PONE-D-21-21989R1) of our manuscript. All of these sentences were in the first version (PONE-D-21-21989)? We already revised all of these sentences. *(Figure 3 title, Result: Trends and projections of the age-standardized incidence rates, paragraph: 2)*
