# Supplementary material for: A rapid rise in hormone receptor-positive and HER2-positive breast cancer subtypes in Southern Thai women: A population-based study in Songkhla
Source: PLoS One. 2022 Mar 28;17(3):e0265417. doi: 10.1371/journal.pone.0265417 (PMC8959182; doi:10.1371/journal.pone.0265417)
Supplement: Attachment — Submitted filename: Response to Reviewers_26022022.docx [file pone.0265417.s006.docx]

**Response to Reviewers**

c*omment on PONE-D-21-21989R3*

We added the information of our data deposit in the Data Archiving and Networking Services (DANS) in subtopic “Population denominators” and “Management of missing data, paragraph 1”, and we added a reference No. 19 in the manuscript as “19. Chuaychai, A. 1000 imputed data set of receptor status for breast cancer. 2021. Data Archiving and Networking Services (DANS). https://doi.org/10.17026/dans-xn5-5286”.

1. Please include captions for your Supporting Information files at the end of your manuscript, and update any in-text citations to match accordingly. Please see our Supporting Information guidelines for more information: http://journals.plos.org/plosone/s/supporting-information.

**Response:** We already added captions of the supporting information files at the end of our manuscript.
